# *BHLHE40* Is a Transcriptional Regulatory Target of *NFE2L3* in Triple-Negative Breast Cancer

**DOI:** 10.32604/or.2025.070793

**Published:** 2026-01-19

**Authors:** Shail Rakesh Modi, Terrick Andey, George Acquaah-Mensah

**Affiliations:** Department of Pharmaceutical Sciences, Massachusetts College of Pharmacy and Health Sciences, Worcester, MA 01608, USA

**Keywords:** Nuclear factor erythroid 2-like 3 (NFE2L3/NRF3), basic helix-loop-helix family member E 40 (BHLHE40/DEC1), triple-negative breast cancer, transcriptional regulatory networks, master regulators

## Abstract

**Objectives:**

The current treatment options and therapeutic targets for triple-negative breast cancer (TNBC), an aggressive subtype of breast cancer (BrCA), are limited. This study aimed to identify novel biomarkers and transcriptional regulatory networks (TRN) inherent in TNBC samples.

**Methods:**

We analyzed pan-cancer BrCA datasets from The Cancer Genome Atlas (TCGA) to compare triple-positive breast cancer (TPBC) with TNBC. TRN algorithms and virtual inference of protein-enriched regulon (VIPER) were used to identify master regulators and their target genes. Utilizing TNBC cells (MDA-MB-231 and MDA-MB-468), we validated the relationship of nuclear factor erythroid 2-like 3 (NFE2L3) and basic helix-loop-helix family member E 40 (BHLHE40) by performing a luciferase assay. The expression levels of these targets were measured after transfections with plasmid and siRNA via qRT-PCR and western blots. The effect of these genes on cell proliferation and migration was studied using phenotypic assays.

**Results:**

Using computational approaches, we identified *NFE2L3* as a master regulator with *BHLHE40* as its target gene. NFE2L3 protein binds to the promoter region of *BHLHE40* and regulates its transcriptional activity. Additionally, silencing and overexpressing *NFE2L3* and *BHLHE40* in TNBC cell lines MDA-MB-231 and MDA-MB-468 showed that *NFE2L3* directly regulates *BHLHE40* at both transcriptional and translational levels. We found that BHLHE40 requires NFE2L3 for cell proliferation and migration in TNBC.

**Conclusion:**

These findings underscore the significance of *NFE2L3* and *BHLHE40* in TNBC, highlighting *NFE2L3’s* role in regulating the oncogenic activity of *BHLHE40* in TNBC cells.

## 1 Introduction

Cancer is characterized by complex interactions among genetic, epigenetic, and environmental factors that perturb cellular homeostasis and drive malignant transformation. Cancer treatment has evolved from non-selective therapies to targeted and immunomodulatory approaches that intervene at specific molecular nodes of tumor progression [1]. Advances in molecular oncology have enabled a deeper understanding of how perturbations in signaling and transcriptional programs can shape cancer phenotypes, paving the way for precision medicine.

Breast cancer (BrCA) is one of the three leading causes of cancer mortality in women worldwide, along with lung cancer and colorectal cancer [2]. According to a report by the American Cancer Society, approximately 2 million breast cancer cases will be diagnosed in 2024, and an estimated 611,720 deaths are expected in the United States of America [3]. Triple-negative breast cancer (TNBC) is one of the most aggressive types of breast cancer that accounts for 15%**–**20% of breast cancer cases [4]. Compared to other types of breast cancer, various challenges are associated with the effective management of TNBC. First, cells from TNBC samples lack the expression of estrogen receptor (ER), progesterone receptor (PR), and lack of overexpression of human epidermal growth factor 2 (HER2) receptor, which limits the use of hormonal therapy or molecularly targeted therapy agents such as tamoxifen, anastrozole, and trastuzumab [5,6]. Secondly, treatment of TNBC involving the use of chemotherapeutic agents is associated with unwanted side effects and the development of resistant phenotypes [7]. Additionally, TNBC has high recurrence and poor overall survival rates [8]. Further, there is a dearth of suitable molecular targets and target-based therapies for the effective treatment of TNBC [9].

Over the years, there has been an exponential growth in biological datasets, with repositories of sequencing data for RNA, DNA, proteins, and epigenetic modifications [10]. These big datasets are useful in addressing various biological concerns, such as phenotyping of patients, drug repurposing, and discovery of biomarkers [11]. Computational methods to identify biomarkers are plenty; however, we focused on the use of transcriptional regulatory network (TRNs) algorithms, such as Algorithm for the Reconstruction of Accurate Cellular Networks (ARACNe), Gene Network Inference with Ensemble of Trees (GENEI3), and the Inferelator, and detect aberrant protein activity using Virtual Inference of Protein-activity by Enriched Regulon analysis (VIPER). The approaches of all three algorithms for creating a TRN are different. ARACNe uses the data processing inequality (DPI) theorem, which determines a mutual information (MI) threshold and ranks the genes based on the MI score for every transcription factor/target [12]. GENEI3 uses a machine learning approach, called random forests, to rank-sort the transcription factor/targets and create a regulatory network [13]. Similarly, the Inferelator uses regression models for inferring the regulatory method [14]. These networks are used by VIPER to predict master regulators, which are the top genes in their hierarchy, based on the differential expression patterns of target genes and the score assigned to them [15,16]. VIPER-based analyses have been instrumental in studies of glioblastoma [17], immune signaling [18] and breast cancer [19]. As demonstrated previously by our lab VIPER, along with ARACNe, led to the identification of differentially expressed patterns in genes between black/African-American and Caucasian patients with breast cancer [20].

To address the above challenges, this study aimed to identify novel biomarkers and transcriptional regulatory networks within TNBC biology by using publicly available datasets to delineate genes and their molecular associates implicated in TNBC development. We investigated TNBC biology and identified several master regulators along with their target genes. One such master regulator was the nuclear factor erythroid 2-related factor 3 (NFE2L3/NRF3), a member of the Cap ‘n’ Collar family of proteins that are linked to several types of cancers, including cancers of the colon, thyroid, pancreas, and kidney [21]. The study hypothesized that NFE2L3 regulates the expression of basic helix-loop-helix family member e40 (*BHLHE40*), a key member in the regulation of the circadian rhythm, which plays an important role in the development of breast cancer, along with other cancers. We characterized the regulatory relationship between NFE2L3 and *BHLHE40* and its impact on cell proliferation and migration in TNBC.

## 2 Materials and Methods

### 2.1 Data Source and Acquisition

The RNA-seq 2016 pan-cancer breast cancer dataset from The Cancer Genome Atlas (TCGA) was downloaded from www.isb-cgc.appspot.com [22]. Expression and phenotype data were accessed and processed using Bioconductor R (Version 4.3.1) packages (‘BigRquery’ [Version 1.5.1] and ‘tidyverse’ [Version 2.0]). Based on the phenotype (immunohistochemistry) data, associated patient samples were divided into two groups: triple-positive breast cancer (TPBC) and TNBC, and corresponding gene expression data were processed. A list of transcription factors that were used as part of the process to reverse-engineer TRNs were identified using annotations from the Gene Ontology (http://geneontology.org/) [23].

### 2.2 Differentially Expressed Genes (DEGs) and Enrichment Analysis

An analysis was performed using a Bioconductor package in R, ‘DESeq2’ (Version 1.48.2), to identify genes expressed differently between the TNBC and TPBC groups as previously described [24,25]. The false discovery rate (FDR) cutoff was set at 5%. The DEGs were represented as a heatmap using the tool, a Bioconductor package in R, ‘ggplot2’ (Version 3.5.2). The DEGs obtained were analyzed for over-represented pathways using a comprehensive R archive network (CRAN) package in R, ‘EnrichR’ (Version 3.4) [26]. Four databases accessed from within EnrichR were used to analyze these DEGs: “GO_biological_process_2021” [23], “KEGG_2021” [27], “Reactome_2022” [28], and “Wikipathways_2021” [29].

### 2.3 Identification of Master Regulators

TRNs were reverse-engineered from the gene expression compendium using ARACNe [12], GENIE3 [13] and the Inferelator algorithm [14]. Regulons were identified using the resulting TRNs. Those regulons subsequently served as input for the Virtual Inference of Protein-activity by Enriched Regulon analysis (VIPER) [15]. VIPER (Version 1.42) was used to infer protein activity and assign a score based on the differential expression of the target genes within the regulon, thus identifying master regulators of regulons driving the differences in gene expression between the TNBC and TPBC groups [20]. Validation of the target genes was performed using Harmonizome 3.0 (https://maayanlab.cloud/Harmonizome). Within Harmonizome, databases such as JASPAR position weighted matrix (PWMs), Encyclopedia of DNA Elements (ENCODE), and ChIP-X Enrichment Analysis (ChEA) were used to validate the TF-target relationships.

### 2.4 Survival Analyses

Survival analyses were investigated using Bioconductor packages in R, survival (Version 3.8), and survminer (Version 0.5.1). Patients were classified based on high expression and low expression of the gene at the level. Mean gene expression levels across patients, along with standard deviations, were calculated. Genes with “high expression” had expression levels higher than the mean by at least 0.5 times the standard deviation. Genes with “low expression” had expression levels lower than the mean by at least 0.5 times the standard deviation.

### 2.5 Visualization of Complex Networks

Cytoscape (Version 3.8.0, Cytoscape Consortium, USA) is an open-source graph (network) visualization tool useful for analyzing molecular interaction networks. We mapped the networks generated from ARACNe, GENIE3, and the Inferelator on Cytoscape and visualized the interactions between each regulator gene and its target genes [30].

### 2.6 Cell Culture and Transfection

MDA-MB-231 (#HTB-26) and MDA-MB-468 (#HTB-132) triple-negative breast cancer cell lines were purchased from American Type Culture Collection (ATCC; Manassas, VA, USA), which were authenticated and free of mycoplasma contamination. These cells were cultured in DMEM/F12 high glucose medium (#A4192102 Thermo Fisher Scientific, Waltham, MA, USA) supplemented with 10% fetal bovine serum (FBS) (#FBS001 Neuromics, Edina, MN, USA), 2% antibiotic-antimycotic solution (#15640055 penicillin (10**–**12 mg/mL)**/**streptomycin (10,000–12,000 U/mL)/amphotericin (25–30 μg/mL) and 500 μL of Fungin (#NC9326704 Thermo Fisher Scientific), and were maintained at 37°C in a humidified incubator containing 5% CO_2_. Expression plasmids for NFE2L3 and BHLHE40 were synthesized using the pcDNA3.1(+) vector and purchased from Genscript Biotech (Piscataway, NJ, USA). Additionally, the BHLHE40 vector was conjugated with the Nanoluc vector and purchased from GenScript Biotech (Piscataway). siRNA for silencing of NFE2L3 (#sc-38107) and BHLHE40 (#sc-106769), along with control siRNA-A (#sc-37007), were purchased from Santa Cruz Biotechnologies (Dallas, TX, USA). Lipofectamine 3000 and p3000 (#L3000015), which were used for transient transfection, were purchased from Thermo Fisher Scientific.

### 2.7 RNA Interference of NFE2L3 and BHLHE40

Three flasks (siControl, NFE2L3 siRNA, and BHLHE40 siRNA) of TNBC cells were cultured in 75 cc flasks and incubated until they attained a confluency of 80%. Each of the lyophilized siRNA NFE2L3 and siRNA BHLHE40 was diluted in 330 μL of RNase-free water to yield a 10 μM concentration. Control siRNA-A was diluted in 66 μL of RNAse-free water to yield a 10 μM concentration. For each group, solution A was prepared by mixing 40 μL of either control siRNA-A (siControl), NFE2L3 siRNA (siNFE2L3), or BHLHE40 siRNA (siBHLHE40) with 650 μL of the siRNA transfection medium. Solution B for each group was prepared by mixing 50 μL of Lipofectamine 3000 (Thermo Fisher Scientific) with an siRNA transfection medium (SantaCruz Biotechnology, Dallas, TX, USA). Solution A (siRNA concentration 0.58 μM) was added to solution B, and the mixture was allowed to sit at room temperature for 45 min. The cells were washed with 2 mL of siRNA transfection medium and aspirated. The mixture was added to the flasks along with 5 mL of siRNA transfection medium with a final concentration of siRNA of 0.063 μM. The mixture was gently spread over the cells, and the flasks were incubated for 6 h in an incubator. After 6 h, the solution in the flasks was aspirated and replaced with fresh medium for 48 h. Experiments were performed in three biological triplicates and were used for subsequent assays.

### 2.8 Overexpression of NFE2L3 and BHLHE40

TNBC Cells were cultured in 25 cc flasks until they reached a confluency of 80%. Two tubes containing 125 μL of plasmid transfection medium were diluted with 10 μL of Lipofectamine 3000 reagent (Solution A). A 10 μg quantity of lyophilized control, *NFE2L3*, or *BHLHE40* plasmids was resuspended in 50 μL of RNAse-free water (Thermo Fisher Scientific), giving a concentration of 0.2 μg/μL. In a separate tube, 10 μg of the plasmid, along with 250 μL of plasmid transfection medium and 15 μL of p3000 (Thermo Fisher Scientific), were mixed (Solution B), diluting the concentration of plasmid to 0.032 μg/μL. A 1:1 ratio of solution A and solution B was mixed, yielding a final concentration of plasmid as 0.016 μg/μL, which was then incubated at room temperature for 15 min. The mixture was spread over the cells and incubated for 3 days. Experiments were performed in three biological triplicates and used for subsequent assays.

### 2.9 RNA Extraction and Quantitative Reverse Transcriptase Polymerase Chain Reaction (qRT-PCR)

Expressions of genes *NFE2L3* and *BHLHE40* following RNA interference and induction of gene expression (as described above) were measured using qRT-PCR. Total RNA from each cell line was extracted using the RNeasy Plus Mini Kit (#74134 Qiagen, Germantown, MD, USA) using the manufacture**r’**s protocol. The RNA was checked for purity and quantified using NanovVue Plus (#30196 GE Healthcare, Chicago, IL, USA). Using the QuantiTect SYBR Green RT-PCR kits (# 204243, Qiagen, Germantown, MD, USA), cDNA was amplified and measured in real-time using Mastercycler Realplex2 (#5345/6300 Eppendorf, Enfield, CT, USA). Primers were purchased from Origene Technologies (Rockville, MD, USA), and the forward and reverse primer sequences for NFE2L3 (#HP207626), BHLHE40 (#HP207148), and GAPDH (#HP205798) are listed below. The expression levels of NFE2L3 and BHLHE40 were normalized with GAPDH values, and fold change was calculated using the 2^**−**ΔΔCt^ method.

**NFE2L3:** 5^′^-CCAGTTGCTTTCATCACAGCCTG-3^′^, 3^′^-CACATCCTGACTTATAGCCTGGC-5^′^ **BHLHE40:** 5^′^-TAAAGCGGAGCGAGGACAGCAA-3^′^, 3^′^-ATGTTCGGGTAGGAGATCCTTC-5^′^ **GAPDH:** 5^′^-GTCTCCTCTGACTTCAACAGCG-3^′^, 3^′^-ACCACCCTGTTGCTGTAGCCAA-5^′^.

### 2.10 Western Blot Analysis

Protein expressions of NFE2L3 and BHLHE40 were measured using Western blot as previously described [31] following RNA interference and induction of gene expression (as described above). Briefly, whole-cell protein lysates were prepared using RIPA Lysis Buffer [62.5 mM Tris-HCl (pH 6.8), 2% SDS, and 10% glycerol] with protease inhibitor according to the manufacturer’s protocol (Thermo Fisher Scientific). Protein concentrations were measured using Pierce^TM^ Bicinchoninic Acid Protein Assay Kit (#23225 Thermo Fisher Scientific) according to the manufacturer’s protocol, and 12.5 μg per lane of total protein was loaded onto an AnyKD Mini-PROTEAN TGX Precast Protein Gel (#4569033 Bio-Rad, Hercules, CA, USA) and separated by SDS-PAGE. Subsequently, proteins were transferred to 0.2 μM nitrocellulose membranes (#1620112 Bio-Rad Laboratories), and the membranes were blocked with 3% Bovine Serum Albumin (BSA; #AC611910100 Fischer Scientific) solution in phosphate-buffered saline with 0.1% Tween-20 (PBST) overnight whilst shaking. The membranes were washed with PBST solution for 5 min three times, and then incubated with primary antibodies against NFE2L3 (rabbit polyclonal antibody; #A09888, Boster Bio, Pleasanton, CA, USA), BHLHE40 (rabbit polyclonal antibody; #ABN1737, Millipore-Sigma, Burlington, MA), and GAPDH (rabbit monoclonal antibody; #2118, Cell Signaling Technology, Danvers, MA, USA), were diluted in blocking solution at 4°C overnight. All primary antibodies were used at dilutions of 1:1000. The membranes were then washed for 5 min three times and incubated with Anti-rabbit IgG secondary antibody (goat monoclonal antibody; #7074, Cell Signaling Technology) at dilutions of 1:2000 at room temperature for 2 h. The membranes were then exposed to a working solution of SuperSignal West Pico PLUS Chemiluminescent Substrate reagent (#34580 Thermo Fisher Scientific) according to the manufacturer’s protocol, and the protein bands were visualized using ImageQuant^TM^ LAS 4000 (GE Healthcare, Chicago, IL, USA). The bands were quantified using ImageJ (Version 1.53k, National Institutes of Health, Bethesda, MD, USA) software and normalized using respective controls.

### 2.11 Dual-Luciferase Reporter Assay

MDA-MB-231 and MDA-MB-468 cells were seeded in a 96-well white opaque plate (1 × 10^4^ cells per well) and incubated overnight. A pGL4.54[luc2/TK] plasmid (Promega Corp, Madison, WI, USA) was used as an internal control as a normalizer for transfection efficiency. Along with the internal control, the cells were transfected with the BHLHE40 plasmid conjugated with the NanoLuc luciferase plasmid. Similarly, to assess the effects of NFE2L3, another set of cells was co-transfected with the NFE2L3 plasmid along with the internal control and the BHLHE40 plasmid conjugated with the NanoLuc luciferase plasmid. The cells were then incubated for 72 h. Luciferase activity was measured using a Nano-Glo Dual-Luciferase reporter assay system (#N1541, Promega), which contained One-Glo EX Luciferase reagent and NanoDLR Stop and Glo reagent according to the manufacturer. The cells were treated with One-Glo EX Luciferase reagent. The plate was incubated on an orbital shaker at 100 RPM for 5 min, and luminescence was measured using a Synergy H1 plate reader (Agilent, Santa Clara, CA, USA). The cells were then treated with NanoDLR Stop and Glo reagent and placed on an orbital shaker for 10 min. The luminescence was measured using a cell plate reader at an integration time of 1 s. The luciferase activity was measured by normalizing the values obtained from the luciferase activity with values obtained from the Stop and Glo Reagent.

### 2.12 Co-Immunoprecipitation Assay

Co-immunoprecipitation (Co-IP) assay was performed as per the manufacture**r’**s protocol [32]. Total protein was extracted from each cell line using RIPA lysis buffer (#87788 Thermo Fisher Scientific) and was incubated with specific primary antibodies (NFE2L3 (#A09888, 1:1000, Boster Bio), BHLHE40 (#ABN1737, 1:1000, Millipore-Sigma) overnight at 4°C. The protein mixture was mixed with Protein A magnetic beads (#73778, Cell Signaling Technology) and incubated at room temperature with rotation for 20 min on a rotary shaker (VWR, Radnor, PA, USA) at 200 RPM. The mixture was washed using the lysis buffer five times while keeping on ice between washes. The mixture was resuspended with SDS sample buffer, followed by vortexing at high speed and heating at 95°C**–**100°C for 5 min to separate the magnetic beads from the immunocomplex. The mixtures were separated using a magnetic rack, which pulled the magnetic beads to the bottom while retaining the analytes (immunocomplexes) in the supernatant. The supernatant was collected and loaded on AnyKD Mini-PROTEAN TGX Precast Protein Gel (Bio-Rad) and subjected to immunoblotting as described under Section 2.7.

### 2.13 Treatment Groups

The phenotypic studies were conducted in MDA-MB-231 cells to assess the effect of genes on cell proliferation and migration. Cells were treated with siRNA of NFE2L3 (siNFE2L3) followed by the addition of NFE2L3 plasmid or BHLHE40 plasmid. Similarly, cells were treated with siRNA of BHLHE40 (siBHLHE40) followed by the addition of NFE2L3 plasmid and BHLHE40 plasmid. A total of nine (9) groups were evaluated for the phenotypic studies, namely, Control, siNFE2L3, siNFE2L3 + NFE2L3 plasmid, siNFE2L3 + BHLHE40 plasmid, siBHLHE40, siBHLHE40 + NFE2L3 plasmid, siBHLHE40 + BHLHE40, NFE2L3 plasmid, and BHLHE40 plasmid. The cells were harvested in 25 cc flasks, and the respective siRNA and plasmid treatments were performed based on the group as previously described.

### 2.14 Cell Proliferation Assay

Following treatment of MDA-MB-231 with the treatment groups as mentioned above, 1 × 10^4^ cells per well were seeded in a 96-well plate format and incubated for 24, 48, and 72 h. The cells were then washed with 1× PBS (pH 7.4), stained with 0.01% Alamar blue (#R7017-1G, Millipore-Sigma), and incubated for 2 h at 37°C. Fluorescence readings were taken using a BioTek Synergy H1 Multimode Reader (Santa Clara) at 530/590 nm. Results were computed as mean ± SD of cell proliferation as a percentage of control.

### 2.15 Migration Assay

Following treatment of MDA-MB-231 with the treatment groups as mentioned above, 5 × 10^4^ cells per well were seeded in a 24-well plate overnight to achieve a cell monolayer formation. A scratch/wound was created across the length of the cell monolayer using a 200 mL pipette tip. The media was removed and replenished with 5% FBS-supplemented media. Brightfield light micrographs of the scratch/wound were acquired using an EVOS M7000 (Invitrogen, Waltham, MA, USA) microscope at an initial time point (0 h), and the cells were incubated for 24, 48, and 72 h. The cells were washed with 1× PBS (pH 7.4) and fixed with 70% ethanol for 15 mins, followed by staining with 1% crystal violet solution. Brightfield images of the scratch/wounds were acquired following the crystal violet staining at the respective time points. The area of the scratch was measured using ImageJ (Version 1.54k, National Institutes of Health), and the wound closure rate was calculated as follows: ([initial scratch area **−** final scratch area]/initial scratch area) × 100. Results were presented as light micrographs and graphs of mean ± standard deviation (SD) of wound closure rate as a percent of baseline.

### 2.16 Statistical Analysis

All experiments were repeated three times independently, and Prism 8.0 (GraphPad Software, San Diego, CA, USA) was used for data analysis. Welch’s *t*-test was employed for the analysis of basal protein expression and luciferase assay. Cell Proliferation, cell migration, gene expression, and protein expression results were analyzed using one-way ANOVA followed by a post-hoc Tukey’s test. A *p*-value less than 0.05 was considered statistically significant.

## 3 Results

### 3.1 The Cell Cycle Processes Were the Overrepresented Pathway for the DEGs between the TNBC and TPBC Groups

**Figure 1:**
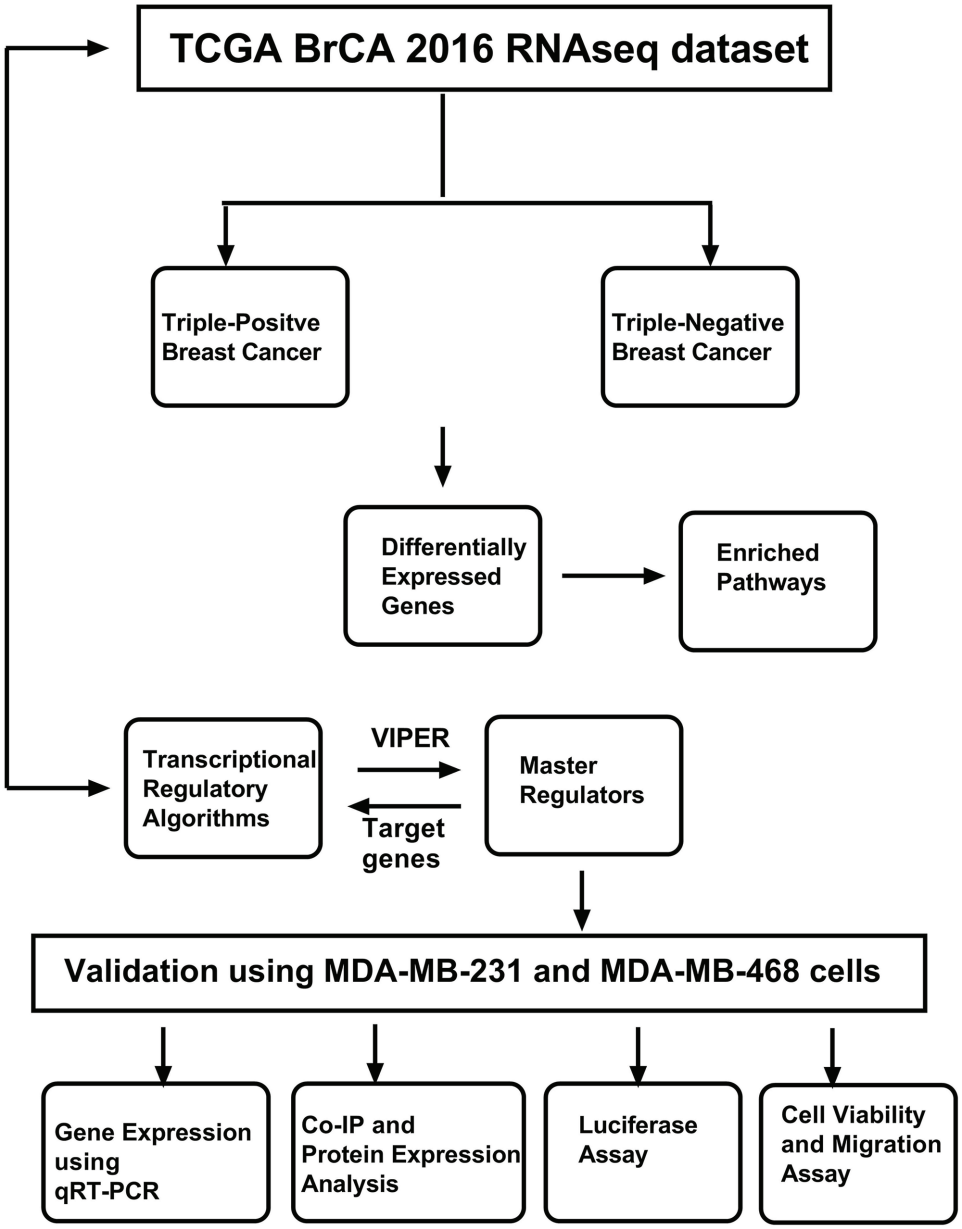
Workflow of the study. The study uses The Cancer Genome Atlas (TCGA) Breast Cancer (BrCA) RNAseq dataset, which was used to identify master regulators using various computational approaches. The identified master regulator and transcriptional regulatory network (TRN) were validated using breast cancer cell lines and cell-based assays. Abb: VIPER: Virtual Inference of Protein-activity by Enriched Regulon analysis; qRT-PCR: Quantitative Reverse Transcription Polymerase Chain Reaction; Co-IP: Co-Immunoprecipitation

**Figure 2:**
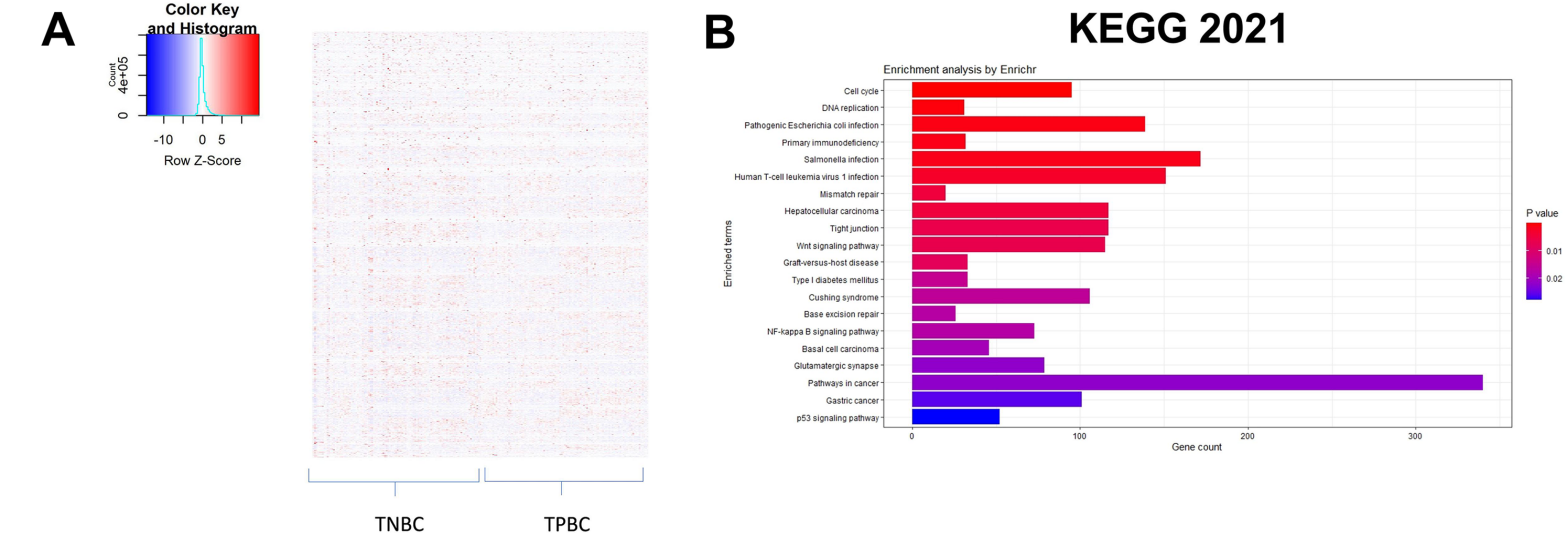

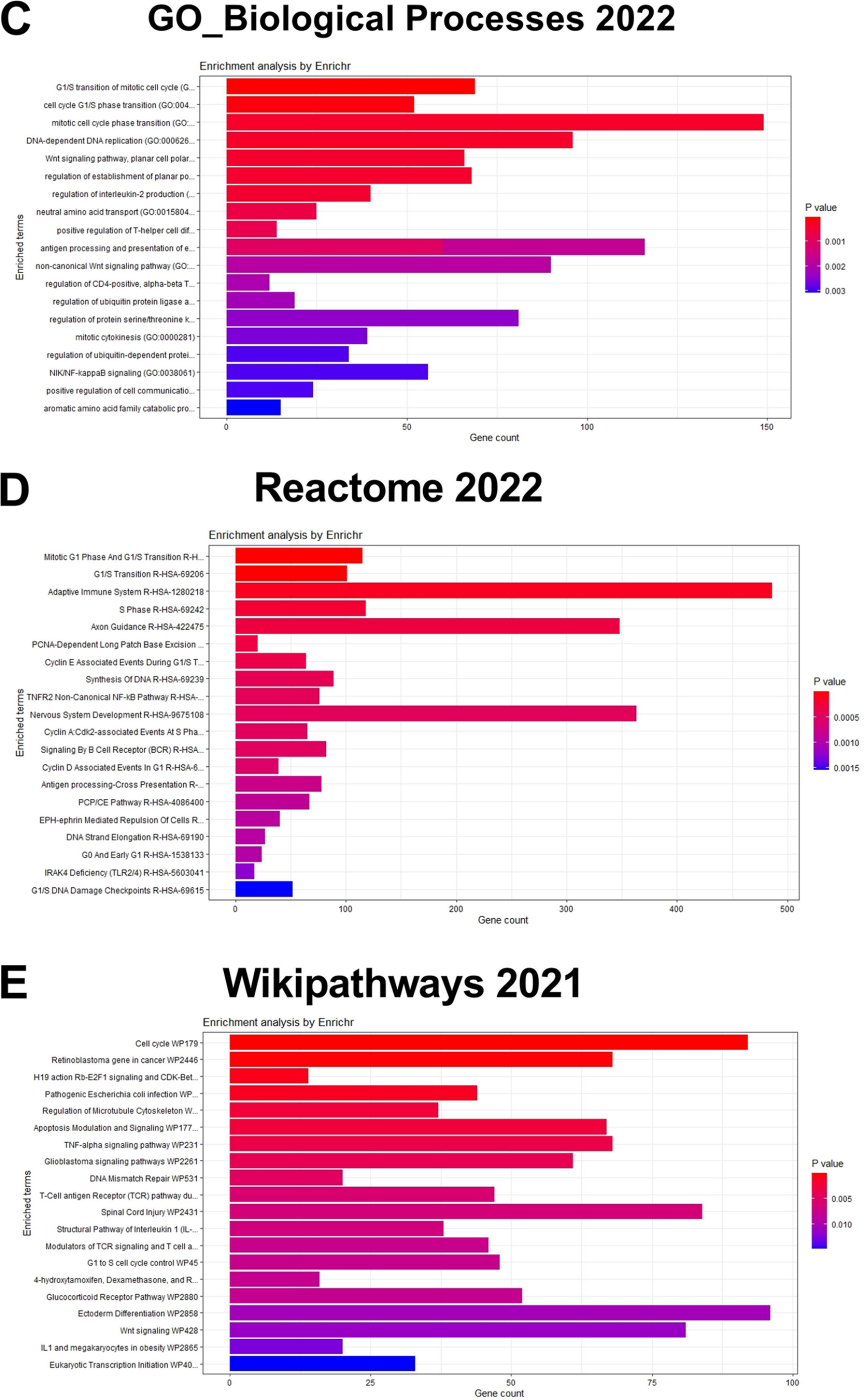
Identification of differentially expressed genes and enriched pathways. (**A**) Heatmap illustrating patterns of differential gene expression between triple-negative breast cancer (TNBC) and triple-positive breast cancer (TPBC) groups. The color key in the top-left corner ranges from blue to red, indicating low to high values. Red boxes indicate proteins that are up-regulated in TNBC compared to TPBC, while blue boxes indicate proteins that are down-regulated in TNBC compared to TPBC. Enrichment plots showing enriched pathways on genes differentially expressed between TPBC and TNBC groups generated using EnrichR using different databases, (**B**) KEGG_2021_human, (**C**) GO_Biological_Process 2022, (**D**) Wikipathway_2021, and (**E**) Reactome_2022. The color coding is based on the *p*-values (key on the right side); red indicates the lowest *p*-value and blue indicates the highest *p*-value. Abbv: KEGG: Kyoto Encyclopedia of Genes and Genomes; GO_Biological_process: Gene Ontology Biological Process

The workflow of the entire study is depicted in Fig. 1. The study used the RNA-seq 2016 pan-cancer breast cancer dataset from TCGA and found approximately 12,000 genes differentially expressed (DEGs) between TPBC and TNBC groups at a False Discovery Rate (FDR) of 5%. Out of the DEGs, 5020 genes were downregulated (log2FC < **−**1, padj < 0.05), and 6923 genes were upregulated (log2FC > 1, padj < 0.05) in TNBC compared to TPBC. The patterns of expression are depicted in a heatmap (Fig. 2A). A negative log2fold change indicated genes that were down-regulated in the TNBC group compared to the TPBC group, while a positive value indicated genes that were up-regulated in the TNBC group compared to the TPBC group. These DEGs were analyzed using four databases within *EnrichR* for over-represented pathways. All these resulting gene sets had the Cell cycle and related processes as the top-enriched pathway with the lowest significant *p*-value and a substantial number of genes (Fig. 2B–E). The DEG list included most of those involved in the cell cycle pathway, either during mitosis or G1, or S phase. Additionally, there were several other pathways, such as the “Wnt signaling pathway”, “NK/NF-kB signaling”, “DNA replication”, “T-cell signaling”, and “DNA mismatch and repair”, in the DEGs that participated across all the datasets. All these pathways contribute to or are connected to cancer cell survival, cell proliferation, and metastasis.

### 3.2 Identification of Master Regulators

**Figure 3:**
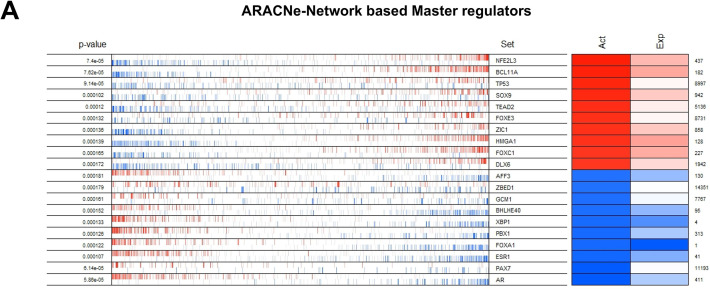

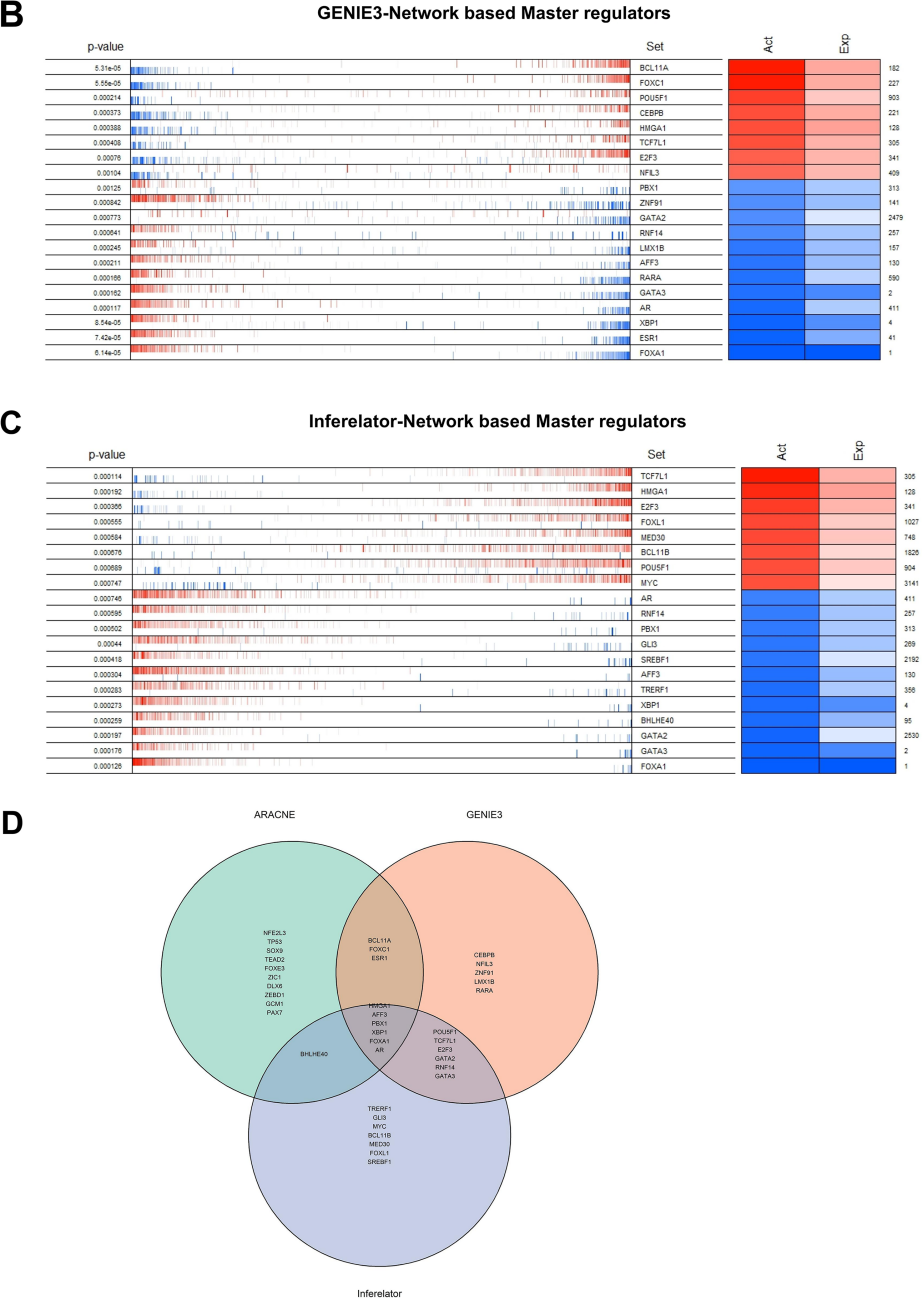
Identification of master regulators. The top twenty differentially active transcription regulators, along with their corresponding regulons enriched (aggregated at the ends of the list of genes ranked by their levels of expression) between TNBC and TPBC groups. (**A**) ARACNe**-**Network Based master regulators, (**B**) GENIE3 Network Based master regulators, (**C**) The Inferelator Network Based master regulators. In the second column, the genes are rank-sorted (left to right) from the most downregulated in the TNBC group (relative to the TPBC group) to the most upregulated, and vertical bars represent targets of the transcription regulators (named in the third column) in the TRNs. Red bars and boxes represent increased expression; blue bars and boxes represent suppressed expression. The fourth and fifth columns represent inferred differential protein activity and gene expression, respectively. (**D**) Venn Diagram depicting common master regulators from the three networks

**Figure 4:**
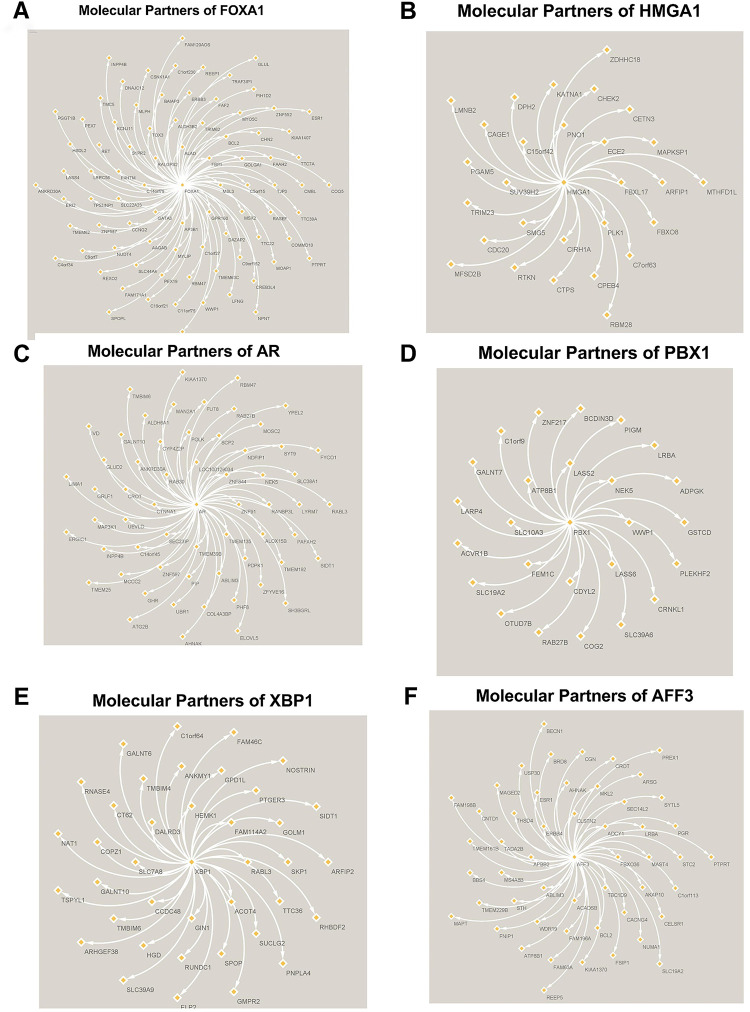
Molecular Partners of six master regulators obtained using transcriptional regulatory networks via VIPER. **(A)** FOXA1, **(B)** HMGA1, **(C)** AR, **(D)** PBX1, **(E)** XBP1 and **(F)** AFF3. VIPER: Virtual Inference of Protein-activity by Enriched Regulon analysis

Three different transcriptional regulatory algorithms were employed to construct regulatory networks, namely, the Algorithm for the Reconstruction of Accurate Cellular Networks (ARACNe), Gene Network Inference with an Ensemble of Trees (GENIE3), and the Inferelator. The network generated by each algorithm, along with the expression file and the list of transcription factors, was analyzed using Virtual Inference of Protein Activity by Enriched-Regulon analysis (VIPER). VIPER calculates a score based on the protein regulon and the differential expression of the target genes between the TNBC and TPBC groups. The top twenty master regulators obtained from each algorithm are depicted in Fig. 3A–C. An intersection of these master regulators is depicted in a Venn Diagram (Fig. 3D). Six consensus master regulators were identified from the three networks: *HMGA1, AFF3, XBP1, PBX1, FOXA1*, and *AR*. Identification of master regulators using ARACNe-derived regulatory networks has been validated and well-documented [15]. In this study, we identified master regulators from regulatory networks derived using ARACNe, GENIE3, and the Inferelator. To validate the common targets of the master regulators, we employed a feature of Harmonizome, a collection of experimentally determined transcription factor datasets such as JASPAR PWMs, ENCODE, and ChEA [33]. These datasets have lists of transcriptional regulators and their corresponding gene targets that are validated using ChIP-seq analysis. Table 1 contains the target genes of FOXA1 that are validated by the JASPAR PWM and ENCODE transcription factor database. Out of the 83 target genes, 55 target genes are reported or validated, leaving 23 target genes that were previously not established as *FOXA1* targets. The target genes may thus be deemed novel regulatory targets of *FOXA1*. Similarly, the regulatory targets of *HMGA1, AR, AFF3, XBP1, and PBX1* are reported in Tables 2–6. The molecular networks are also depicted in Fig. 4.

**Table 1:** Validation of target genes of FOXA1 using the Harmonizome database

Target	ARACNe	GENIE3	The Inferelator	Validated
C9orf152	Yes	Yes	Yes	JASPAR and ENCODE
ERBB3	Yes	Yes	Yes	ENCODE
NUDT4	Yes	Yes	Yes	ENCODE
MOAP1	Yes	Yes	Yes	Novel
TRAF3IP1	Yes	Yes	Yes	ENCODE
LASS4	Yes	Yes	Yes	Novel
ESR1	Yes	Yes	Yes	JASPAR and ENCODE
GPR160	Yes	Yes	Yes	ENCODE
MYLIP	Yes	Yes	Yes	ENCODE
TRIM62	Yes	Yes	Yes	ENCODE
AAGAB	Yes	Yes	Yes	ENCODE
TJP3	Yes	Yes	Yes	ENCODE
KCNJ11	Yes	Yes	Yes	Novel
C19orf21	Yes	Yes	Yes	Novel
CMBL	Yes	Yes	Yes	ENCODE
CSNK1A1	Yes	Yes	Yes	ENCODE
C9orf7	Yes	Yes	Yes	Novel
INPP4B	Yes	Yes	Yes	ENCODE
ALAD	Yes	Yes	Yes	ENCODE
MSX2	Yes	Yes	Yes	ENCODE
S1PR2	Yes	Yes	Yes	Novel
RBM47	Yes	Yes	Yes	ENCODE
MYO5C	Yes	Yes	Yes	JASPAR
TP53INP1	Yes	Yes	Yes	Novel
LFNG	Yes	Yes	Yes	ENCODE
ZNF552	Yes	Yes	Yes	ENCODE
PEX7	Yes	Yes	Yes	Novel
PTPRT	Yes	Yes	Yes	Novel
C4orf34	Yes	Yes	Yes	Novel
GATA3	Yes	Yes	Yes	ENCODE
GOLGA1	Yes	Yes	Yes	ENCODE
SLC22A23	Yes	Yes	Yes	ENCODE
TTC22	Yes	Yes	Yes	Novel
BAIAP3	Yes	Yes	Yes	ENCODE
REXO2	Yes	Yes	Yes	ENCODE
COMMD10	Yes	Yes	Yes	ENCODE
REEP1	Yes	Yes	Yes	Novel
ERI2	Yes	Yes	Yes	ENCODE
GLUL	Yes	Yes	Yes	ENCODE
MSL3	Yes	Yes	Yes	Novel
C1orf27	Yes	Yes	Yes	ENCODE
ALDH3B2	Yes	Yes	Yes	ENCODE
SLC44A4	Yes	Yes	Yes	Novel
FAAH2	Yes	Yes	Yes	ENCODE
RET	Yes	Yes	Yes	Novel
C11orf75	Yes	Yes	Yes	Novel
TTC7A	Yes	Yes	Yes	Novel
DNAJC12	Yes	Yes	Yes	JASPAR and ENCODE
ASB8	Yes	Yes	Yes	ENCODE
PGGT1B	Yes	Yes	Yes	ENCODE
RALGPS2	Yes	Yes	Yes	ENCODE
C5orf15	Yes	Yes	Yes	ENCODE
P4HTM	Yes	Yes	Yes	Novel
TMEM63C	Yes	Yes	Yes	Novel
FAF2	Yes	Yes	Yes	JASPAR
ZNF587	Yes	Yes	Yes	JASPAR
CREB3L4	Yes	Yes	Yes	ENCODE
PIH1D2	Yes	Yes	Yes	Novel
HSDL2	Yes	Yes	Yes	ENCODE
COQ5	Yes	Yes	Yes	JASPAR and ENCODE
SPOPL	Yes	Yes	Yes	ENCODE
AP3B1	Yes	Yes	Yes	ENCODE
BCL2	Yes	Yes	Yes	ENCODE
CCNG2	Yes	Yes	Yes	ENCODE
RASEF	Yes	Yes	Yes	ENCODE
MLPH	Yes	Yes	Yes	ENCODE
FAM171A1	Yes	Yes	Yes	Novel
TTC39A	Yes	Yes	Yes	ENCODE
C1orf230	Yes	Yes	Yes	Novel
TMEM62	Yes	Yes	Yes	Novel
FAM120AOS	Yes	Yes	Yes	JASPAR and ENCODE
FBP1	Yes	Yes	Yes	ENCODE
DAZAP2	Yes	Yes	Yes	JASPAR and ENCODE
TOX3	Yes	Yes	Yes	JASPAR and ENCODE
PEX19	Yes	Yes	Yes	JASPAR and ENCODE
CHN2	Yes	Yes	Yes	ENCODE
LRRC56	Yes	Yes	Yes	ENCODE
WWP1	Yes	Yes	Yes	ENCODE
KIAA1407	Yes	Yes	Yes	ENCODE
TMC5	Yes	Yes	Yes	ENCODE
NPNT	Yes	Yes	Yes	ENCODE
ANKRD30A	Yes	Yes	Yes	JASPAR
C14orf79	Yes	Yes	Yes	ENCODE

Note: ARACNe, Algorithm for the Reconstruction of Accurate Cellular Networks; GENIE3, Gene Network Inference with Ensemble of Trees; JASPAR, JASPAR Annotated Sequence Profiles for Analysis of Regulation; ENCODE, Encyclopedia of DNA Elements.

**Table 2:** Validation of target genes of AR using the Harmonizome database

Target	ARACNe	GENIE3	The Inferelator	Validated
ECE2	Yes	Yes	Yes	Novel
SUV39H2	Yes	Yes	Yes	Novel
CTPS	Yes	Yes	Yes	Novel
FBXO8	Yes	Yes	Yes	Novel
CETN3	Yes	Yes	Yes	Novel
DPH2	Yes	Yes	Yes	Novel
TRIM23	Yes	Yes	Yes	Novel
RTKN	Yes	Yes	Yes	Novel
LMNB2	Yes	Yes	Yes	Novel
RBM28	Yes	Yes	Yes	Novel
PLK1	Yes	Yes	Yes	Novel
PNO1	Yes	Yes	Yes	Novel
SMG5	Yes	Yes	Yes	Novel
CPEB4	Yes	Yes	Yes	Novel
ARFIP1	Yes	Yes	Yes	Novel
CHEK2	Yes	Yes	Yes	Novel
CAGE1	Yes	Yes	Yes	Novel
CDC20	Yes	Yes	Yes	Novel
ZDHHC18	Yes	Yes	Yes	Novel
MFSD2B	Yes	Yes	Yes	Novel
MTHFD1L	Yes	Yes	Yes	Novel
FBXL17	Yes	Yes	Yes	Novel
C15orf42	Yes	Yes	Yes	Novel
CIRH1A	Yes	Yes	Yes	Novel
C7orf63	Yes	Yes	Yes	Novel
MAPKSP1	Yes	Yes	Yes	Novel
KATNA1	Yes	Yes	Yes	Novel
PGAM5	Yes	Yes	Yes	Novel

Note: ChEA: Chip-X Enrichment Analysis.

**Table 3:** Validation of target genes of AR using the Harmonizome database

Target	ARACNe	GENIE3	The Inferelator	Validated
SLC38A1	Yes	Yes	Yes	ChEA
ALDH6A1	Yes	Yes	Yes	Novel
MCCC2	Yes	Yes	Yes	ChEA
FYCO1	Yes	Yes	Yes	ChEA
LIMA1	Yes	Yes	Yes	Novel
ZNF844	Yes	Yes	Yes	Novel
TMEM39B	Yes	Yes	Yes	Novel
RANBP3L	Yes	Yes	Yes	Novel
CYP4Z2P	Yes	Yes	Yes	Novel
ZNF597	Yes	Yes	Yes	Novel
ZFYVE16	Yes	Yes	Yes	ChEA
MOSC2	Yes	Yes	Yes	Novel
GLUD2	Yes	Yes	Yes	Novel
ELOVL5	Yes	Yes	Yes	Novel
RBM47	Yes	Yes	Yes	ChEA
TMEM25	Yes	Yes	Yes	Novel
RAB30	Yes	Yes	Yes	ChEA
ABLIM3	Yes	Yes	Yes	ChEA
NDFIP1	Yes	Yes	Yes	ChEA
CROT	Yes	Yes	Yes	ChEA
UBR1	Yes	Yes	Yes	Novel
PAFAH2	Yes	Yes	Yes	ChEA
FUT8	Yes	Yes	Yes	ChEA
MAP3K1	Yes	Yes	Yes	Novel
SIDT1	Yes	Yes	Yes	ChEA
TMBIM6	Yes	Yes	Yes	Novel
TMEM135	Yes	Yes	Yes	ChEA
SEC23IP	Yes	Yes	Yes	Novel
ALOX15B	Yes	Yes	Yes	Novel
POLK	Yes	Yes	Yes	Novel
C14orf45	Yes	Yes	Yes	Novel
PHF8	Yes	Yes	Yes	Novel
SYT9	Yes	Yes	Yes	Novel
GALNT10	Yes	Yes	Yes	Novel
AHNAK	Yes	Yes	Yes	ChEA
YPEL2	Yes	Yes	Yes	Novel
ERGIC1	Yes	Yes	Yes	ChEA
LOC100129034	Yes	Yes	Yes	Novel
PIP	Yes	Yes	Yes	ChEA
NEK5	Yes	Yes	Yes	Novel
ANKRD30A	Yes	Yes	Yes	ChEA
GHR	Yes	Yes	Yes	ChEA
TMEM192	Yes	Yes	Yes	Novel
RAB27B	Yes	Yes	Yes	ChEA
GRLF1	Yes	Yes	Yes	Novel
SH3BGRL	Yes	Yes	Yes	ChEA
KIAA1370	Yes	Yes	Yes	Novel
ATG2B	Yes	Yes	Yes	ChEA
CTNNA1	Yes	Yes	Yes	ChEA
PDPK1	Yes	Yes	Yes	Novel
SCP2	Yes	Yes	Yes	ChEA
UEVLD	Yes	Yes	Yes	Novel
COL4A3BP	Yes	Yes	Yes	ChEA
LYRM7	Yes	Yes	Yes	Novel
MAN2A1	Yes	Yes	Yes	ChEA
INPP4B	Yes	Yes	Yes	Novel
RABL3	Yes	Yes	Yes	ChEA
IVD	Yes	Yes	Yes	Novel
ZNF91	Yes	Yes	Yes	Novel

Note: ChEA: Chip-X Enrichment Analysis.

**Table 4:** Validation of target genes of AFF3 using the Harmonizome database

Target	ARACNe	GENIE3	The Inferelator	Validated
FBXO36	Yes	Yes	Yes	Novel
ABLIM3	Yes	Yes	Yes	Novel
AKAP10	Yes	Yes	Yes	Novel
AHNAK	Yes	Yes	Yes	Novel
STH	Yes	Yes	Yes	Novel
FSIP1	Yes	Yes	Yes	Novel
PGR	Yes	Yes	Yes	Novel
BRD8	Yes	Yes	Yes	Novel
BBS4	Yes	Yes	Yes	Novel
PREX1	Yes	Yes	Yes	Novel
REEP5	Yes	Yes	Yes	Novel
CLSTN2	Yes	Yes	Yes	Novel
FAM196A	Yes	Yes	Yes	Novel
LRBA	Yes	Yes	Yes	Novel
THSD4	Yes	Yes	Yes	Novel
ATP8B1	Yes	Yes	Yes	Novel
CELSR1	Yes	Yes	Yes	Novel
ARSG	Yes	Yes	Yes	Novel
MAGED2	Yes	Yes	Yes	Novel
FNIP1	Yes	Yes	Yes	Novel
FAM198B	Yes	Yes	Yes	Novel
TBC1D9	Yes	Yes	Yes	Novel
APBB2	Yes	Yes	Yes	Novel
CACNG4	Yes	Yes	Yes	Novel
MKL2	Yes	Yes	Yes	Novel
MS4A8B	Yes	Yes	Yes	Novel
KIAA1370	Yes	Yes	Yes	Novel
STC2	Yes	Yes	Yes	Novel
CGN	Yes	Yes	Yes	Novel
TMEM161B	Yes	Yes	Yes	Novel
PTPRT	Yes	Yes	Yes	Novel
MAPT	Yes	Yes	Yes	Novel
ADCY1	Yes	Yes	Yes	Novel
ACADSB	Yes	Yes	Yes	Novel
MAST4	Yes	Yes	Yes	Novel
ESR1	Yes	Yes	Yes	Novel
WDR19	Yes	Yes	Yes	Novel
NUMA1	Yes	Yes	Yes	Novel
SYTL5	Yes	Yes	Yes	Novel
USP30	Yes	Yes	Yes	Novel
TMEM229B	Yes	Yes	Yes	Novel
BECN1	Yes	Yes	Yes	Novel
SLC19A2	Yes	Yes	Yes	Novel
ERBB4	Yes	Yes	Yes	Novel
BCL2	Yes	Yes	Yes	Novel
SEC14L2	Yes	Yes	Yes	Novel
TADA2B	Yes	Yes	Yes	Novel
FAM63A	Yes	Yes	Yes	Novel
C1orf113	Yes	Yes	Yes	Novel
CROT	Yes	Yes	Yes	Novel
CNTD1	Yes	Yes	Yes	Novel

**Table 5:** Validation of target genes of XBP1 using the Harmonizome database

Target	ARACNe	GENIE3	The Inferelator	Validated
HEMK1	Yes	Yes	Yes	Novel
GIN1	Yes	Yes	Yes	Novel
TTC36	Yes	Yes	Yes	Novel
GPD1L	Yes	Yes	Yes	Novel
COPZ1	Yes	Yes	Yes	Novel
ELP2	Yes	Yes	Yes	Novel
ARFIP2	Yes	Yes	Yes	Novel
FAM46C	Yes	Yes	Yes	Novel
RNASE4	Yes	Yes	Yes	Novel
ARHGEF38	Yes	Yes	Yes	Novel
RABL3	Yes	Yes	Yes	Novel
SLC7A8	Yes	Yes	Yes	Novel
SPOP	Yes	Yes	Yes	Novel
GOLM1	Yes	Yes	Yes	Novel
TMBIM4	Yes	Yes	Yes	Novel
TMBIM6	Yes	Yes	Yes	Novel
PNPLA4	Yes	Yes	Yes	Novel
SIDT1	Yes	Yes	Yes	Novel
C1orf64	Yes	Yes	Yes	Novel
NAT1	Yes	Yes	Yes	Novel
SLC39A9	Yes	Yes	Yes	Novel
FAM114A2	Yes	Yes	Yes	Novel
CCDC48	Yes	Yes	Yes	Novel
SUCLG2	Yes	Yes	Yes	Novel
PTGER3	Yes	Yes	Yes	Novel
CT62	Yes	Yes	Yes	Novel
HGD	Yes	Yes	Yes	Novel
RHBDF2	Yes	Yes	Yes	Novel
NOSTRIN	Yes	Yes	Yes	Novel
GALNT6	Yes	Yes	Yes	Novel
TSPYL1	Yes	Yes	Yes	Novel
ACOT4	Yes	Yes	Yes	Novel
DALRD3	Yes	Yes	Yes	Novel
RUNDC1	Yes	Yes	Yes	Novel
SKP1	Yes	Yes	Yes	Novel
ANKMY1	Yes	Yes	Yes	Novel
GALNT10	Yes	Yes	Yes	Novel
GMPR2	Yes	Yes	Yes	Novel

**Table 6:** Validation of target genes of PBX1 using the Harmonizome database

Target	ARACNe	GENIE3	The Inferelator	Validated
LARP4	Yes	Yes	Yes	Novel
OTUD7B	Yes	Yes	Yes	Novel
NEK5	Yes	Yes	Yes	Novel
SLC10A3	Yes	Yes	Yes	Novel
CDYL2	Yes	Yes	Yes	Novel
COG2	Yes	Yes	Yes	Novel
CRNKL1	Yes	Yes	Yes	Novel
GSTCD	Yes	Yes	Yes	Novel
LRBA	Yes	Yes	Yes	Novel
BCDIN3D	Yes	Yes	Yes	Novel
C1orf9	Yes	Yes	Yes	Novel
ACVR1B	Yes	Yes	Yes	Novel
LASS6	Yes	Yes	Yes	Novel
LASS2	Yes	Yes	Yes	Novel
FEM1C	Yes	Yes	Yes	Novel
RAB27B	Yes	Yes	Yes	Novel
SLC39A6	Yes	Yes	Yes	Novel
PLEKHF2	Yes	Yes	Yes	Novel
ADPGK	Yes	Yes	Yes	Novel
PIGM	Yes	Yes	Yes	Novel
ZNF217	Yes	Yes	Yes	Novel
GALNT7	Yes	Yes	Yes	Novel
SLC19A2	Yes	Yes	Yes	Novel
WWP1	Yes	Yes	Yes	Novel
ATP8B1	Yes	Yes	Yes	Novel
LARP4	Yes	Yes	Yes	Novel
OTUD7B	Yes	Yes	Yes	Novel
NEK5	Yes	Yes	Yes	Novel
SLC10A3	Yes	Yes	Yes	Novel
CDYL2	Yes	Yes	Yes	Novel
COG2	Yes	Yes	Yes	Novel
CRNKL1	Yes	Yes	Yes	Novel
GSTCD	Yes	Yes	Yes	Novel
LRBA	Yes	Yes	Yes	Novel
BCDIN3D	Yes	Yes	Yes	Novel
C1orf9	Yes	Yes	Yes	Novel
ACVR1B	Yes	Yes	Yes	Novel
LASS6	Yes	Yes	Yes	Novel
LASS2	Yes	Yes	Yes	Novel
FEM1C	Yes	Yes	Yes	Novel
RAB27B	Yes	Yes	Yes	Novel
SLC39A6	Yes	Yes	Yes	Novel
PLEKHF2	Yes	Yes	Yes	Novel
ADPGK	Yes	Yes	Yes	Novel
PIGM	Yes	Yes	Yes	Novel
ZNF217	Yes	Yes	Yes	Novel
GALNT7	Yes	Yes	Yes	Novel
SLC19A2	Yes	Yes	Yes	Novel
WWP1	Yes	Yes	Yes	Novel
ATP8B1	Yes	Yes	Yes	Novel

### 3.3 NFE2L3: Master Regulator and Its Target Genes

**Figure 5:**
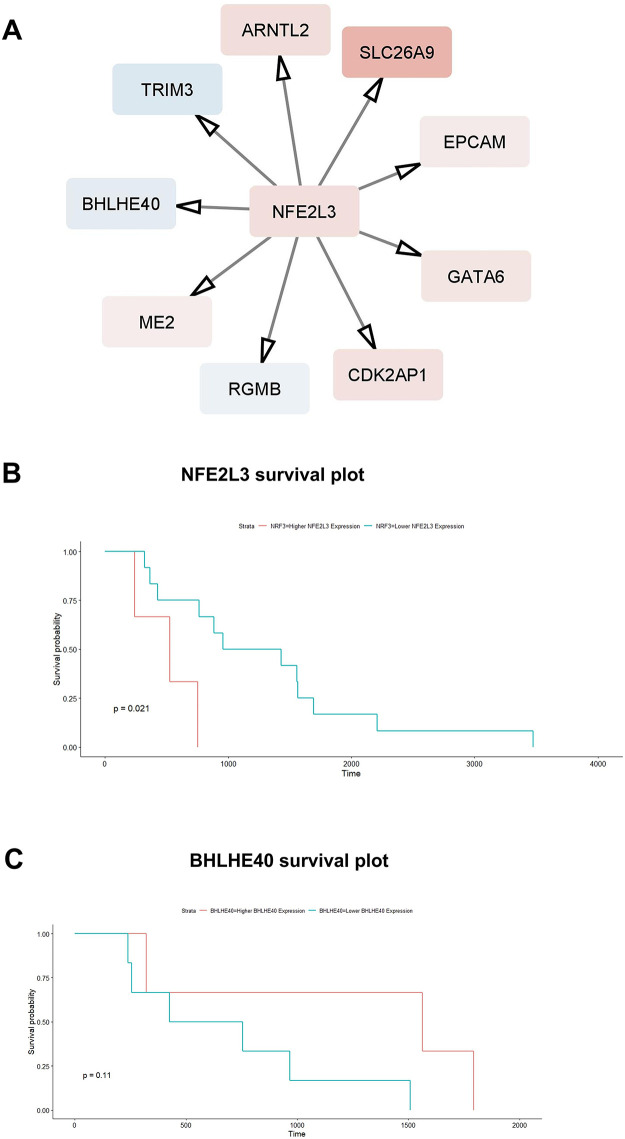
(**A**) Consensus regulatory targets of NFE2L3, based on the regulatory networks inferred using GENIE3 and ARACNe. The red represents the protein upregulated in TNBC compared to TPBC. The blue represents the protein downregulated in TNBC compared to TPBC. Kaplan-Meier survival plots of **(B)** NFE2L3 and **(C)** BHLHE40 using the TCGA RNA-seq Breast cancer dataset. The red line represents higher expression of the gene, while the blue line represents lower expression of the gene. *p* < 0.05. Higher NFE2L3 expression corresponds to poor overall survival than those with low NFE2L3 expression, while there is no significant difference in survival between patients with high and low expressions of BHLHE40

NFE2L3 was identified as one of the master regulators from the ARACNe-generated network, which had the smallest *p*-value and had increased expression in the TNBC group compared to the TPBC group. The regulatory target genes of NFE2L3 were more than 100 in each generated network. To limit and confirm the validity of target genes, we combined the regulatory target genes of NFE2L3, which were in common between ARACNe and GENIE3, limiting to nine targets depicted in Fig. 5A, along with their differential expression patterns between TNBC and TPBC. The nine consensus target genes of NFE2L3 have, separately, been extensively researched with respect to breast cancer. The finding that NFE2L3 regulates the expression of each of these nine target genes together is a novel result of this study. The transcriptional regulatory relationship between NFE2L3 and BHLHE40, one of the nine consensus targets of NFE2L3, became our focus of experimental validation using cell line models. The expression levels of BHLHE40 were downregulated in the TNBC group, while the expression levels of NFE2L3 were upregulated in the TNBC group compared to the TPBC group. BHLHE40 is known to promote cell proliferation and migration in breast cancer [34,35]. Surprisingly, BHLHE40 expression levels in TNBC were suppressed relative to those in TPBC. This led to the question of whether NFE2L3 regulates the expression of BHLHE40 in TNBC. Fig. 5B,C represent**s** the survival analysis of the high and low expression of NFE2L3 and BHLHE40 using the TCGA BrCA dataset. High expression of NFE2L3 is inversely correlated to overall survival, which means patients with high expression of NFE2L3 have poor overall survival compared to patients with lower expression of NFE2L3. On the other hand, there is no significant difference in survival rates between patients with high or low expression of BHLHE40.

### 3.4 NFE2L3 Regulates BHLHE40 Expression at the Transcriptional Level

**Figure 6:**
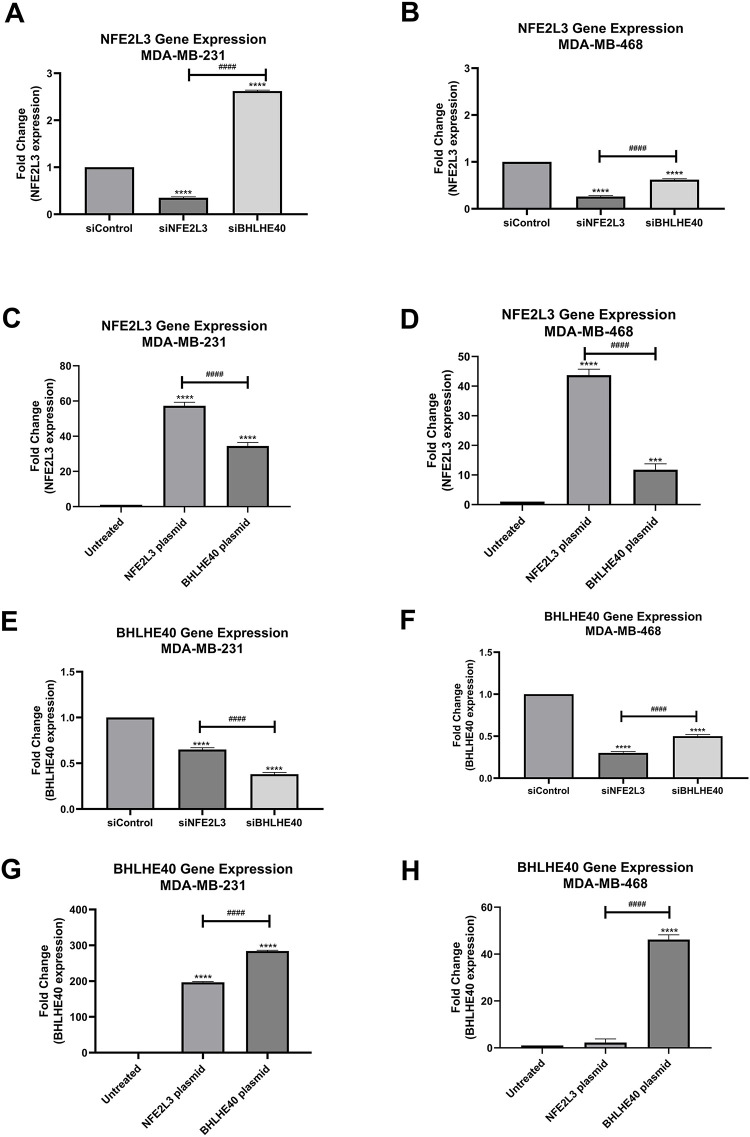
Gene expression quantification using quantitative Reverse Transcriptase polymerase chain reaction (qRT-PCR). NFE2L3 gene expression in (**A**) MDA-MB-231 and (**B**) MDA-MB-468 cells in siControl, siNFE2L3, and siBHLHE40 groups. (**C**,**D**) Gene expression in (**C**) MDA-MB-231 and (**D**) MDA-MB-468 cells in untreated, NFE2L3 plasmid and BHLHE40 plasmid groups. BHLHE40 gene expression using qRT-PCR. BHLHE40 gene expression in (**E**) MDA-MB-231 and (**F**) MDA-MB-468 cells following treatment with siControl, siNFE2L3, and siBHLHE40. BHLHE40 gene expression in (**G**) MDA-MB-231 and (**H**) MDA-MB-468 cells following treatment with vehicle (untreated), NFE2L3 plasmid, and BHLHE40 plasmid. One-way ANOVA followed by post-hoc Tukey’s test was used. Compared to the siControl or untreated group: ***indicates *p* < 0.001, and ****indicates *p* < 0.0001. Compared to siNFE2L3 or NFE2L3 plasmid group: ^####^indicates *p* < 0.0001

Triple-negative breast cancer cell lines (MDA-MB-231 and MDA-MB-468) were used to validate siRNA and plasmids targeting *NFE2L3* (siNFE2L3 and NFE2L3 plasmid, respectively) and *BHLHE40* (siBHLHE40 and BHLHE40 plasmid, respectively). Treatment with siNFE2L3 significantly decreased *NFE2L3* mRNA expression levels in both cell lines compared to the control group (Fig. 6A,B). Notably, *NFE2L3* mRNA expression was downregulated by 35% and 26% in the MDA-MB-231 and MDA-MB-468 siNFE2L3-treated cell groups, respectively, compared to the control group. Paradoxically, NFE2L3 levels were increased in MDA-MB-231 when treated with siBHLHE40 compared to the control (Fig. 6A). In contrast, treatment with the NFE2L3 plasmid significantly increased *NFE2L3* mRNA expression levels in both cell lines compared to the untreated control group. *NFE2L3* mRNA expression levels were upregulated by 57.28% and 43.71% compared to the control in MDA-MB-231 and MDA-MB-468 in *NFE2L3* plasmid-treated cells, respectively (Fig. 6C,D). The mRNA expression levels of *BHLHE40* exhibited decreased by 38% and 50% in MDA-MB-231 and MDA-MB-468 cells treated with siBHLHE40, respectively, compared to the control group. Similarly, *BHLHE40* mRNA expression decreased by 65% and 30% in MDA-MB-231 and MDA-MB-468 cells treated with siNFE2L3, respectively, compared to the control. In both cell lines, *BHLHE40* mRNA expression was significantly reduced in cells treated with siNFE2L3 and siBHLHE40 compared to the control group (Fig. 6E,F). Conversely, a substantial increase of 284- and 47-fold in *BHLHE40* expression levels was observed in MDA-MB-231 and MDA-MB-468 cells treated with the *BHLHE40* plasmid, respectively. These levels were significantly higher compared to the control group in both cell lines (Fig. 6G,H). Furthermore, the expression levels of *BHLHE40* were significantly elevated in MDA-MB-231 and MDA-MB-468 cells treated with *NFE2L3* by 196% and 3.94%, respectively, suggesting a transcriptional relationship between *NFE2L3* and *BHLHE40*.

### 3.5 NFE2L3 Regulates BHLHE40 Expression at the Translational Level

The basal protein expression of NFE2L3 and BHLHE40 was assessed via Western blotting, with GAPDH serving as a loading control. The normalized NFE2L3 protein levels were 54.72 ± 1.86% and 60.76 ± 0.88% in MDA-MB-231 and MDA-MB-468 cells, respectively. The normalized BHLHE40 protein levels were 57.49 ± 0.69% and 48.70 ± 0.22% in MDA-MB-231 and MDA-MB-468 cells, respectively. The NFE2L3 basal protein levels were observed to be higher in MDA-MB-468 cells compared to MDA-MB-231 cells, whereas BHLHE40 basal protein expression was higher in MDA-MB-231 cells compared to MDA-MB-468 cells (Figures not added).

**Figure 7:**
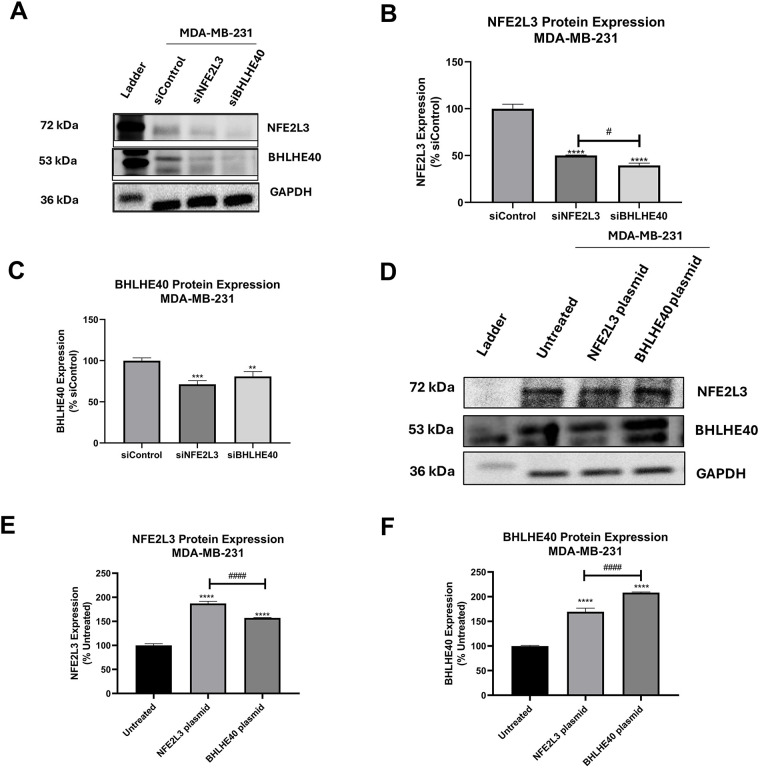
Basal protein expression of NFE2L3 and BHLHE40 NFE2L3 and BHLHE40 in breast cancer cells. (**A**) Representative blots of NFE2L3 and BHLHE40 protein expression following treatment with siControl, siNFE2L3, and siBHLHE40. GAPDH was used as a loading control. (**B**) Graphical presentation of NFE2L3 protein expression in MDA-MB-231 cells following treatment as described. (**C**) Graphical presentation of BHLHE40 protein expression in MDA-MB-231 cells following treatment as described. (**D**) Representative blots of NFE2L3 and BHLHE40 protein expression following treatment with vehicle (Untreated), NFE2L3 DNA plasmid, and BHLHE40 DNA plasmid. GAPDH was used as a loading control. (**E**) Graphical presentation of NFE2L3 protein expression in MDA-MB-231 cells following treatment as described. (**F**) Graphical presentation of BHLHE40 protein expression in MDA-MB-231 cells following treatment as described. One-way ANOVA followed by post-hoc Dunnet’s test was used. Compared to siControl: **indicates *p* < 0.01, ***indicates *p* < 0.001, and ****indicates *p* < 0.0001. Compared to the siNFE2L3 group or NFE2L3 plasmid group: ^#^indicates *p* < 0.05, and ^####^indicates *p* < 0.0001

The protein expression of NFE2L3 and BHLHE40 was determined following siRNA treatment, and the micrographs of the protein blots are presented in Fig. 7A. The protein expression results are representative of only MDA-MB-231 cells, as the results from MDA-MB-468 cells were inconsistent. Treatment with siNFE2L3 resulted in a 50.01 ± 0.56% expression in NFE2L3 protein levels in MDA-MB-231 cells (Fig. 7B). This silencing of NFE2L3 also led to a significant decrease in BHLHE40 protein levels compared to the siControl group, in MDA-MB-231 cells (Fig. 7C). Interestingly, treatment with siBHLHE40 resulted in a significant decrease in NFE2L3 expression in MDA-MB-231 (Fig. 7B).

Additionally, treatment with siBHLHE40 resulted in reduced expression of BHLHE40 protein levels of MDA-MB-231 cells (40.73 ± 1.56%) (Fig. 7C). Notably, both siNFE2L3 and siBHLHE40 treatments significantly reduced BHLHE40 protein expression compared to the siControl group, suggesting an intricate interplay between NFE2L3 and BHLHE40 genes and/or protein.

Transfection with the NFE2L3 plasmid led to a significant increase in the protein levels of both NFE2L3 and BHLHE40 compared to the control group in both cell lines. Representative blots depicting the protein expression of NFE2L3 and BHLHE40 in the NFE2L3 plasmid and BHLHE40 plasmid groups are shown in Fig. 7D. Treatment with the NFE2L3 plasmid resulted in a significant increase in NFE2L3 protein levels in MDA-MB-231 cells (118 ± 1.38%) (Fig. 7E). Similarly, the protein levels of BHLHE40 in NFE2L3-treated cells were significantly increased to 112 ± 1.96% in MDA-MB-231 (Fig. 7F). Moreover, the protein levels of BHLHE40 in BHLHE40-treated cells were also significantly increased in MDA-MB-231 cells (177.12 ± 4.80%). Notably, treatments with the NFE2L3 plasmid resulted in increased BHLHE40 protein expression in MDA-MB-231 cells (Fig. 7F). Overall, induction of NFE2L3 gene expression led to an increase in BHLHE40 mRNA and protein levels, while silencing NFE2L3 gene expression resulted in a decrease in BHLHE40 mRNA and protein levels.

### 3.6 NFE2L3 Binds to the Promoter Region of BHLHE40 and Has a Physical Protein-Protein Interaction

**Figure 8:**
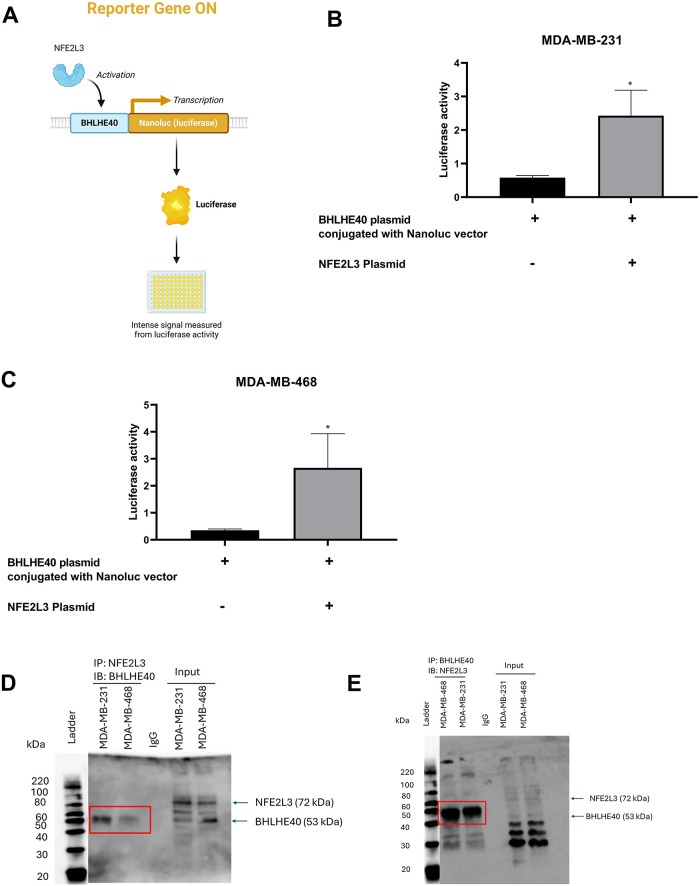
Validation of regulatory relationship between NFE2L3 and BHLHE40. (**A**) Visual representation of luciferase assay with BHLHE40 conjugated with Nanoluc vector, activated by NFE2L3, resulting in production of luciferase activity. Qualitative analysis of BHLHE40 transcriptional activation by NFE2L3 plasmid compared to BHLHE40 plasmid conjugated with the Nanoluc vector in (**B**) MDA-MB-31 and (**C**) MDA-MB-468 cells. Co-immunoprecipitation analysis of NFE2L3 protein and BHLHE40 protein interaction. (**D**) Co-IP of NFE2L3 pulldown with immunoblotting with BHLHE40. (**E**) Co-IP of BHLHE40 pulldown with immunoblotting with NFE2L3. Red boxes indicate the interacting bands. Student’s *t*-test was used to compare the two groups, *indicates *p* < 0.05

To evaluate the activation of the BHLHE40 transcriptional activity, a luciferase-based reporter assay was performed in MDA-MB-231 and MDA-MB-468 cells. Cells were transfected with a BHLHE40 DNA plasmid fused with a luciferase reporter gene and compared with co-transfection of the NFE2L3 DNA plasmid (Fig. 8A). Compared to the BHLHE40 plasmid group, transfection with the NFE2L3 plasmid significantly increased luciferase activity by 2.42- and 2.66-fold in MDA-MB-231 and MDA-MB-468 cells, respectively (Fig. 8B,C). These results suggest that the NFE2L3 protein can bind to the promoter region of the BHLHE40 gene to regulate its transcriptional activity. Co-immunoprecipitation analysis revealed that in both cell lines, there was a physical protein-protein interaction between NFE2L3 and BHLHE40. Based on the blots (Fig. 8D,E), we observed a weak interaction in the MDA-MB-468 cells compared to the MDA-MB-231 cells. Also, the negative isotype control did not show any band at the protein level, which indicates that the antibodies used for NFE2L3 and BHLHE40 do not bind to any off-target proteins. The whole cell lysate was added to identify the protein of interest and other bands.

### 3.7 BHLHE40 in the Absence of NFE2L3 Results in a Decrease in Cell Proliferation and Cell Migration

**Figure 9:**
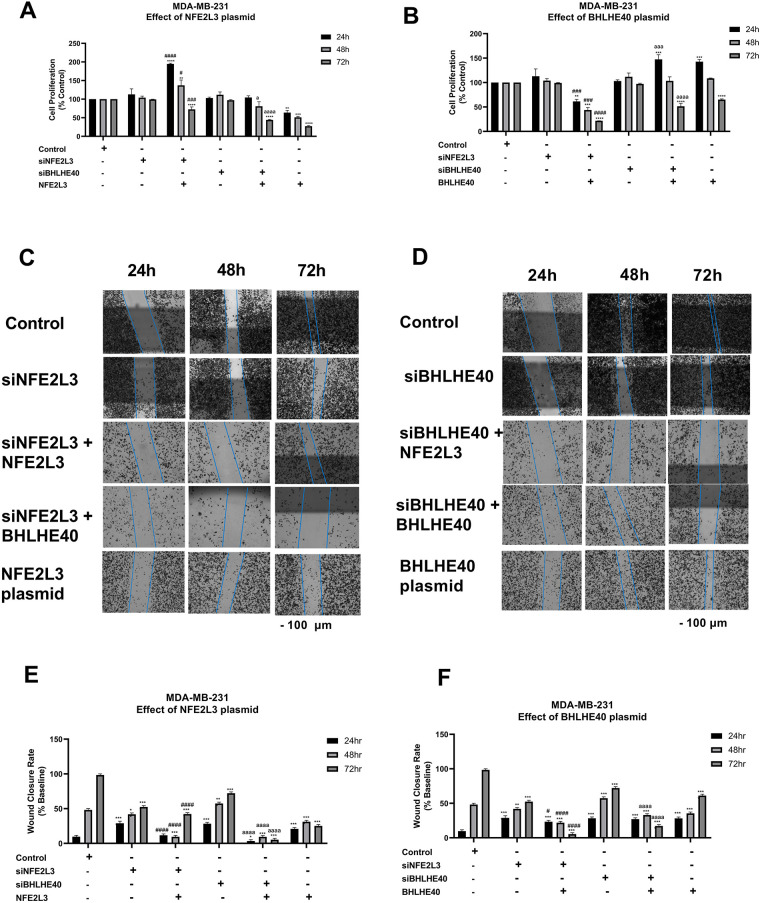
Phenotypic studies on MDA-MB-231 cells. Effect of knockdown or induction of NFE2L3 and BHLHE40 gene expression on MDA-MB-231 cell proliferation. MDA-MB-231 cell**s’** proliferation was determined following treatment with vehicle (Control), siNFE2L3, siBHLHE40, as well as (**A**) NFE2L3 DNA plasmid, or (**B**) BHLHE40 DNA plasmid for 24, 48, and 72 h. Effect of knockdown or induction of NFE2L3 and BHLHE40 gene expression on MDA-MB-231 cell migration. MDA-MB-231 cell migration was determined following treatment with vehicle (Control), siNFE2L3, siBHLHE40, as well as (**C**) NFE2L3 DNA plasmid, or (**D**) BHLHE40 DNA plasmid for 24, 48, and 72 h. (Scale**—**100 μm) (**E**,**F**) Graphical presentation of the estimation of wound closure area following treatment as described above. One-way ANOVA followed by post-hoc Tukey’s test was used. Compared to untreated: *indicates *p* < 0.05, **indicates *p* < 0.01, ***indicates *p* < 0.001, and ****indicates *p* < 0.0001. Compared to siNFE2L3: ^#^indicates *p* < 0.05, ^###^indicates *p* < 0.001, and ^####^indicates *p* < 0.0001. Compared to siBHLHE40, ^a^indicates *p* < 0.05, ^aaa^indicates *p* < 0.001, and ^aaaa^indicates *p* < 0.0001

In MDA-MB-231 cells, the effects of NFE2L3 and BHLHE40 overexpression on cell proliferation were investigated (Fig. 9A,B). Treatment with siNFE2L3 followed by NFE2L3 DNA plasmid administration resulted in significantly increased cell proliferation at 24 h (194 ± 1.83%), followed by a decrease at 48 h (137.99 ± 14.3%), and a significant reduction at 72 h (72.61 ± 6.71%), compared to the control and siNFE2L3 groups (Fig. 9A). Similar trends were observed when the NFE2L3 plasmid was introduced into the siBHLHE40 group. Increases in cell proliferation were observed at 24 h (104.38 ± 4.74%), followed by decreases at 48 h (80.38 ± 12.2%) and significant reductions at 72 h (45.33 ± 0.2%) compared to the control and siBHLHE40 groups (Fig. 9A). Treatment with the BHLHE40 DNA plasmid in cells pre-treated with siNFE2L3 significantly decreased the cell proliferation rate at all time points, compared to the control and siNFE2L3 groups. At 24 h, the cell proliferation was 61.36 ± 4.24% followed by a decrease at 48 h to 43.70 ± 5.199%, and 21.78 ± 0.11% at 72 h (Fig. 9B). Similarly, while the addition of the BHLHE40 plasmid to cells pretreated with siBHLHE40 resulted in an increase in cell proliferation at 24 h followed by a decrease at 48 h, and a significant decrease at 72 h of 51.41 ± 5.08%, compared to control and siBHLHE40 groups (Fig. 9B).

In MDA-MB-231 cells, the effects of NFE2L3 and BHLHE40 overexpression on cell migration were investigated (Fig. 9C,D). Brightfield images of the wound closure are represented in Fig. 9C,D. Treatment with siNFE2L3 followed by NFE2L3 DNA plasmid administration resulted in significantly decreased cell migration at 24 h (11.65 ± 1.8%), followed by a gradual decrease at 48 h (9.57 ± 1.4%), and a significant decrease at 72 h (42.3 ± 1.6%), compared to the siNFE2L3 groups (Fig. 9E). Similar trends were observed when the NFE2L3 plasmid was introduced into the siBHLHE40 group. A decrease in cell migration was observed at 24 h (1.42 ± 1.6%), followed by decreases at 48 h (9.44 ± 1.2%) and significant reductions at 72 h (5.33 ± 1.7%) compared to the siBHLHE40 groups (Fig. 9E).

The treatment of the BHLHE40 DNA plasmid in cells pre-treated with siNFE2L3 significantly decreased the cell proliferation rate at all time points, compared to the siNFE2L3 group. At 24 h, the cell migration was 23.3 ± 1.7% followed by a decrease at 48 h to 21.85 ± 1.99%, and 3.85 ± 1.6% at 72 h (Fig. 9F). Similarly, while the addition of the BHLHE40 plasmid to cells pretreated with siBHLHE40 did not result in an increase in cell migration at 24 and 48 h, at 72 h, there was a significant decrease of 19.21 ± 1.6%, compared to control and siBHLHE40 groups (Fig. 9F).

## 4 Discussion

The aggressive nature of triple-negative breast cancer (TNBC) and the limited availability of targeted treatment options underscore the complexity of breast cancer pathophysiology, highlighting the need for further research and attention. To address these limitations, the study used a 2016 pan-cancer TCGA RNA-seq breast cancer dataset [36,37] to understand the biology of TNBC by identifying those regulatory relationships that play an important role in the development of TNBC. The TCGA dataset is a comprehensive study including multi-omics data across various tumor types, which is ideal to study molecular pathways and mechanisms in cancer. The study identified genes with differential expression in the TNBC and TPBC groups. Most of the DEGs were enriched in cell cycle processes across four robust databases, implying a strong connection with cancer cell survival, proliferation, and migration [38]. Additionally, products of these DEGs participate in pathways related to cancer, such as WNT signaling, NK/NF-ĸB signaling, and p53 signaling. These canonical pathways are well-discussed in their roles in cancer biology [39].

TRNs are hierarchies of relationships between transcription factors (proteins) and their target genes [40,41]. These regulatory networks were reverse-engineered from gene expression compendia with the use of three algorithms: ARACNe, GENIE3, and the Inferelator. ARACNe-generated master regulators via VIPER have been validated in different studies [15,20]. A recent study used the TCGA dataset along with ARACNe and VIPER, identifying master regulators associated with tumor invasiveness [42]. However, the use of GENIE3 and Inferelator-generated master regulators via VIPER is a novel part of this study. The intersection of the common genes led to the identification of six novel master regulators, *HMGA1, AFF3, XBP1, PBX1, FOXA1*, and *AR*, with the addition of their target genes. The individual role of these genes with respect to breast cancer has been discussed in the literature [43–48]. The target gene list that we obtained was validated with target genes present in JASPAR PWM, ENCODE, and ChEA, identifying many novel targets that require validation.

NFE2L3, a master regulator identified using the ARACNe-based network, was studied in triple-negative breast cancer. NFE2L3 is overexpressed in breast cancer, and its role is not completely understood [49]. The role of NFE2L3 in breast cancer is ambiguous and is reportedly both a tumor promoter [50] and a tumor suppressor [51]. Based on the regulatory networks of NFE2L3 within different algorithms, we identified nine common targets of NFE2L3, out of which we decided to explore the NFE2L3/BHLHE40 axis in breast cancer. We posited that targeting of BHLHE40—a pro-metastasis factor in breast cancer—could be affected via targeting NFE2L3 at the upstream of the axis, with resultant regulation of the former and its downstream gene targets. Several recent studies have utilized VIPER in reconstructing transcriptional hierarchies, identifying master regulators such as MYC, STAT3, and FOXM1 as key oncogenic drivers in TNBC [52]. In the broader context of breast cancer transcription regulation, our findings on NFE2L3 and BHLHE40 fit into an emerging paradigm of hierarchical master regulators’ control over tumor state transitions. Identification of NFE2L3 as a potential upstream regulator of BHLHE40 suggests that this axis may constitute part of an unrecognized transcriptional hierarchy in TNBC.

The transfection studies, including knockdown and overexpression of NFE2L3 and BHLHE40 in TNBC cells, supported an interplay between NFE2L3 and BHLHE40 at transcription and translation levels. Generally, induction of NFE2L3 increased the levels of BHLHE40, whereas knockdown of NFE2L3 decreased the expression of BHLHE40 in MDA-MB-231 cells. Dual-luciferase assay and co-immunoprecipitation assay validate the regulatory relationship of NFE2L3 and BHLHE40. The Co-IP shows physical interaction between NFE2L3 and BHLHE40. The results in Fig. 8D,E appear to show an absence of NFE2L3 (IP) and BHLHE40 (IP), following blotting for BHLHE40 and NFE2L3, respectively. A logical explanation for this has to do with the fact that pulling down of the composite proteins may result in a relatively heavier protein complex, depending on the molecular weights of the associated proteins and their relative abundance, and in some cases, resulting in protein bands occurring between the molecular band regions of the associated proteins. It follows that the bands for NFE2L3 and BHLHE40 in Fig. 8D,E, respectively, were not detectable separately because they exist as composites with the other protein and were consequently detected between band regions of the two proteins [53]. While the scope of Co-IP was not extended to characterize the nature of PPI (stable vs. transient, weak vs. strong), the existence of such an interaction between the two proteins points to a possible collaboration in a biological function.

Lastly, the effect of these genes on cell proliferation and cell migration, which are important hallmarks of cancer, was observed using phenotypic assays. While neither knockdown of NFE2L3 nor BHLHE40 was observed to affect MDA-MB-231 cell proliferation over 72 h, knockdown of NFE2L3 followed by induction of BHLHE40 as well as knockdown of BHLHE40 with induction of NFE2L3, were associated with significant decreases in cell proliferation. Interestingly and quite paradoxically, knockdown of BHLHE40—a tumor promoter—followed by its rescue, as well as knockdown of NFE2L3—a tumor suppressor—followed by its rescue, were associated with significant increases in cell proliferation within 24 h and a return to basal levels at 48 h, followed by decreased proliferation at 72 h. And while the tumor suppressor effect of NFE2L3 was observed following its induction after knockdown of BHLHE40 with decreased cell proliferation across all time points, the tumor promoter effect of BHLHE40 was absent following its induction and after knockdown of NFE2L3. While these observations seemed stochastic and paradoxical, it became logical when interpreted through the dependency of BHLHE40 on the transcriptional regulatory influence of NFE2L3 and a possible feedback relationship between them. Notably, whereas the induction of BHLHE40 did not promote cell proliferation over the long term and actually resulted in decreased cell proliferation, induction of NFE2L3 with or without knockdown of either NFE2L3 or BHLHE40 was associated with decreased cell proliferation. Notably, knockdown of NFE2L3 followed by induction of BHLHE40 resulted in decreased cell proliferation, potentially supporting a positive feedback loop mechanism. The cell proliferation dynamics were consistent with those observed with cell migration, where induction of NFE2L3 with or without BHLHE40 knockdown was associated with decreased migratory potential, and more so in the BHLHE40 knockdown group, but also observed with knockdown of NFE2L3 followed by induction of BHLHE40. Altogether, the results provide compelling evidence demonstrating NFE2L3 and BHLHE40 to be engaged in co-regulating the TNBC cancer phenotype. While the exact mechanism of co-regulation of cell proliferation and migration is not yet fully elucidated, results from the luciferase assay, which confirmed NFE2L3 to be a transcriptional regulator of *BHLHE40*, coupled with the likelihood of a feedback loop mechanism, may explain this interplay. BHLHE40 has been linked to the PI3K/AKT pathway and upregulates AKT phosphorylation and promotes oncogenesis. Further studies focused on interrogating the role of NFE2L3 on a hypothetical BHLHE40/PI3K/AKT axis and its downstream targets could open avenues for exploring the clinical relevance of these biologic molecules.

As previously mentioned, past studies demonstrated the conflicting function of NFE2L3 as both a tumor suppressor and promoter and thus, necessitating additional investigation of NFE2L3 across a broader range of cell lines and tissues [50,51]. Currently, there are no approved therapies in the market or in clinical trials targeting NFE2L3 or BHLHE40. Direct inhibition of these targets is only possible due to the use of siRNA, shRNA, and CRISPR. Studies have reported that GSK3 (glycogen synthase kinase 3) phosphorylates NFE2L3; therefore, GSK3 inhibitors such as lithium chloride might affect NFE2L3 expression, which needs to be experimentally investigated [54]. A number of different biological pathways and molecules such as NF-ĸB, WNT/β-catennin pathway, and CDK1 associated with NFE2L3 have been reported across different cancers, [55–57]. Similarly, BHLHE40 interacts with numerous proteins, such as CLOCK-BMAL1, HDAC1/2, SREBF1, and regulates the function of tumor-infiltrating lymphocytes (TILs) and immune checkpoints such as PD1 and CTLA-4 [34,58]. A recent study reported that fatostatin, an SREBF1 inhibitor, suppressed the growth of pancreatic cells with high expression of BHLHE40 via ferroptosis [59]. Indirect inhibition of NFE2L3 and BHLHE40 via these connected pathways or targets is an interesting approach that requires further investigation. Importantly, successfully delineating and exploiting the tumor suppressor effect of NFE2L3 and its direct regulatory influence on BHLHE40 could provide a novel approach to abrogating the tumor-promoting effect of BHLHE40 as a therapeutic strategy against TNBC.

A limitation of this study is using a single dataset; predictions may not be generalizable to other datasets. Using these models, which mainly capture transcriptional regulation, often skips post-transcriptional modifications that influence these genes. Additionally, a single siRNA was used to knock down the expression of these genes. Typically, multiple siRNAs are employed to minimize potential off-target effects. Unfortunately, authors were constrained in using more than a single siRNA due to limited success in obtaining and custom-designing plasmids. Additionally, successive Western blot analysis MDA-MB-468 cells following knockdown and induction of NFE2L3 and BHLHE40 yielded inconsistent results making it challenging to drawn conclusive inferences for this cell line. The results presented in Fig. 7, therefore, represented replicable data for MDA-MB-231 cells only. Authors recognize the reliance on one cell line as a potential limitation to the study. However, authors are confident that the conclusive results obtained from gene-reporter and co-immunoprecipitation studies in MDA-MB-468 cells add rigor and validate the study a high degree. Our findings are based on computational approaches and *in vitro* analysis. Further validation using animal models such as TNBC xenografts or patient-derived models would be essential to establish the physiological and therapeutic significance of this NFE2L3/BHLHE40 axis.

In conclusion, the current study provides important insights into breast cancer biology by identifying novel master regulators and their target genes. Additionally, the NFE2L3/BHLHE40 axis represents a novel pathway in breast cancer development and may potentially hold promise as a therapeutic option.

## Data Availability

Data will be available on request.
